# On forward inferences of fast and slow readers. An eye movement study

**DOI:** 10.1038/srep08432

**Published:** 2015-02-13

**Authors:** Stefan Hawelka, Sarah Schuster, Benjamin Gagl, Florian Hutzler

**Affiliations:** 1 Centre for Cognitive Neuroscience, University Salzburg, Hellbrunnerstr. 34, 5020 Salzburg, Austria

## Abstract

Unimpaired readers process words incredibly fast and hence it was assumed that top-down processing, such as predicting upcoming words, would be too slow to play an appreciable role in reading. This runs counter the major postulate of the predictive coding framework that our brain continually predicts probable upcoming sensory events. This means, it may generate predictions about the probable upcoming word during reading (dubbed forward inferences). Trying to asses these contradictory assumptions, we evaluated the effect of the predictability of words in sentences on eye movement control during silent reading. Participants were a group of fluent (i.e., fast) and a group of speed-impaired (i.e., slow) readers. The findings indicate that fast readers generate forward inferences, whereas speed-impaired readers do so to a reduced extent - indicating a significant role of predictive coding for fluent reading.

The present eye movement study investigated the role of predicting upcoming words during silent reading and the potential differences between fast and slow readers in this cognitive process. We plunge into the topic with an analogy to spoken language comprehension: The speed of speech is fast (~6 syllables per second; Ref. 1). Thus, it was argued that predicting the speaker's upcoming utterance can not facilitate comprehension on behalf of the listener (e.g., Ref. 2). Silent reading is even faster. It proceeds at rates of up to 500 words per minute. After a period of about two decades (around the 1970ies), in which reading was considered a “psycholinguistic guessing game” (e.g., Ref. 3), theories about reading adopted the view that reading is primarily a bottom-up process – similar to theories of speech perception (e.g., Ref. 4). To illustrate, the lexical quality hypothesis of reading^5^,^6^ postulates that lexical representations of words are characterized by the coupling between the words' orthography (i.e., the visual word form), its phonology (the sound of the word) and its meaning (i.e., semantics). However, for skilled (i.e., fast) readers word recognition is considered to proceed from the activation of the orthographic representation towards the activation of phonology and semantics without notable influence from semantic on orthographic processing. In other words, there is little top-down processing in reading. This unidirectional bottom-up process was termed “context-free decoding” – referring to the activation of meaning from the visual percept of a word before a context-based prediction about the word's identity can be generated^7^,^8^.

Following the lexical quality hypothesis, this would mean that the predictability of words in sentences has little influence on the processing times of fast readers (or merely a rather small and late influence during the semantic integration of the words into the sentence context; see below). Another important prediction of the hypothesis is that context affects fast and slow readers differently. The hypothesis states that fast readers possess well specified (high-quality) lexical representations (enabling fast bottom-up processing and hence context-free decoding). For less proficient (slow) readers, the assumption of the hypothesis is that their slower reading rate is due to underspecified lexical representations. Specifically, it is the orthographic and the phonological representations which are supposed to be impaired – preventing fast bottom-up processing. Thus, in slow readers, top-down, context-based processing has the time to wield influence. As a consequence, slow readers benefit from sentential context^9^.

That sentential context has little effect on fast readers contradicts the view of our brain as a *proactive* organ – a view which has become increasingly prominent in the last decade. Proactive means (quite simplified) that our brain generates expectancies of future events. This view is known as the predictive coding framework^10^. In short, the predictive coding framework postulates that every biological system – including our brain – adheres to the principle of trying to minimize “surprise” (e.g., neuronal excitation; Ref. 11). This is accomplished by generating “*forward inferences*” (also “*active inferences*”; Ref. 12) of probable upcoming sensory events. These inferences (or predictions) can be based on prior experiences (e.g., anticipating the appearance of a target in response to a cue dependent on the validity of previous cues; Ref. 13). Concerning object recognition, the fast processing of partial visual information accounts for predictive coding. According to Bar and colleagues^14^, the low spatial frequencies (LSF) of an object are projected rapidly to an anterior brain region (i.e., the orbitofrontal cortex). Then, predictions are generated on the basis of the LSF which pre-activate – top-down – probable objects *before* the bottom-up stream arrives at the respective brain region (i.e., the inferior temporal lobe).

In line with the predictive coding account, there was a paradigmatic shift in the perspective on the influence of top-down generated predictions in language processing. Nowadays, there is a broad agreement that predictions play a significant role. A wealth of evidence, that listeners predict upcoming utterances, was provided by electrophysiological studies (i.e., EEG; e.g., Ref. 15, 16) and by eye movement studies (assessing anticipatory looking; e.g., Ref. 17, 18). The role of *forward inferences* in reading has, as yet, been studied primarily from a “macro-perspective”, that is, for narrative comprehension (e.g., Ref. 19). In such studies participants usually read whole paragraphs and inferential processing is inferred from the processing times of sentences (rather than single words). The empirical evidence of these studies is mixed, but it seems that low-skill readers draw inferences less automatic (i.e., slower) than high-skill readers (e.g., Ref. 19, 20; in this context “skill” refers to global reading ability including vocabulary and comprehension). This finding concurs with an early suggestion Kintsch^21^ made, implying that high reading skill enables automatic access to context-based inferences, whereas readers with lower reading skills must do so in a controlled (i.e., non-automatic) manner.

Two recent neuroimaging studies took a more “nuclear” view by assessing the effect of the predictability of single words in sentences^22^,^23^. Note, however, that they did not assess the predictability effect during “natural” reading, but for the serial, one-by-one presentation of the words of sentences. Their findings concur with the expectation from the predictive coding framework. One of the studies reported a very early effect of word predictability at a posterior brain region, that is, at 50–90 ms after the presentation of high-predictable compared to unpredictable words^22^. Notably, the effect is almost as early as visual information reaches the primary visual cortex (the minimum of the retina-brain lag lies within 40–60 ms; e.g., Ref. 24). The only explanation for such an early effect of predictability probably is that generating a forward inference about the likely identity of a (predictable) word pre-activated a visual template of the word. The other study showed that reading predictable compared to unpredictable words requires less brain metabolism in the visual cortex (i.e., in the occipital lobe; Ref. 23). Furthermore, a recent eye movement study showed that predictability does not require a large amount of preceding context to build-up, but can arise very quickly on the basis of bits of circumscribed, local information (i.e., a single prediction-inducing adjective is sufficient to elicit an effect on the skipping probability of an upcoming noun; Ref. 25).

These recent studies provide strong evidence that upcoming words are indeed predicted; a perspective dubbed *prediction view* in the research domain of language comprehension in order to distinguish it from the *integration view* (e.g., Ref. 16). The latter perspective assumes that the effect of predictability arises late, that is, during the integration of a word in the sentence context (easier and hence faster for predictable words). From the prediction view, one can infer the following (interrelated) expectancies with regard to (silent) reading. First, predictable words are frequently skipped (i.e., they do not receive a fixation). This is indeed a well-documented fact (reviewed in Ref. 26): During reading, about 30% of the words do not receive a fixation (i.e., are skipped) and word predictability is a main factor accounting for the phenomenon. However, current theories of eye movement control do not (directly) acknowledge that *predicting* the identity of upcoming words is the driving force of word skipping (see Discussion). Second, predicting upcoming words may lead to elevated fixation times on words preceding predictable words (because the generation of predictions takes a certain amount of time). Thus, one would expect elevated fixation times prior to word skippings, if these skippings are based on a forward inference about the identity of upcoming words. For this expectation, the existing findings are inconsistent (e.g., Ref. 27; see Discussion). However, strong evidence that the predictability of an upcoming word has an effect *before* the word is encountered was provided by a study of Kliegl and colleagues^28^. The study reported (for an impressively large sample; *n* = 222) *prolonged* fixations on words preceding high-predictable words (which were not skipped). The effect could not be attributed to visually preprocessing the upcoming word, because it was evident even when the visual quality of the preview of the upcoming word was decreased (unlike, e.g., the effect of word frequency). Thus, the authors interpreted the prolongation of fixations in case of predictable upcoming words as evidence that the sentence context made it possible: “*to retrieve word n + 1 from memory*” (*p*. 29; “word n + 1” refers to the upcoming word). They termed this process “*cued memory retrieval*”. In the present study we will, however, retain the term *forward inference* from the predictive coding framework.

## The present study

We assessed the effect of the predictability of (upcoming) words during silent reading with the same sentences as the study by Kliegl and colleagues^28^, that is, the sentences from the Potsdam corpus^29^ which provides predictability norms for each word. Predictability refers to the probability (*p*; range: 0–1) with which a word can be guessed on the basis of the preceding sentence context. Our participants were fast readers and speed-impaired (i.e., slow) readers. Of specific interest will be:

1. The difference in the effect of word predictability between the slow and the fast readers.
2. The relation of the effect of predictability with the individual reading rate of our participants.
3. The time-course of the predictability effect.

*Ad* (1.), for the fast readers, we expect to replicate the original finding, that is, prolonged fixation duration on words preceding predictable compared to unpredictable words^28^. For the slow readers, we may expect that this effect is substantially subdued, if the reported reading-skill related differences in inferential processing for narrative comprehension (i.e., less automatic in low-skill readers) generalizes to visual word recognition on sentence-level. Surprisingly, a previous eye movement study found small effects of reading proficiency for processing predictable versus unpredictable words^30^. The authors concluded that the differences between proficient and less proficient readers are merely “*ones of degree rather than type*” (*p*. 1066). If we find significant differences in the extent to which fast and slow readers generate forward inferences during reading, then the difference might not be merely one of “*degree*”, but a more fundamental discrepancy - accounting, at least in part, for the slow readers' speed impairment. Accordingly, it may be that (*ad* 2.) the most reading-speed impaired participants exhibit the least effect of the predictability of word_n+1_ (our index for the generation of *forward inferences*). On the other hand, they may exhibit the most profound facilitation by the predictability of word_n_. The hypothesized reason is that (*ad* 3.) word predictability exerts a late effect in the slow readers, because of less automatized inferential processing^21^.

## Methods

### Participants

We assessed the eye movements of a total of 70 participants (including those of Ref. 31). In both groups (i.e., the fast and the slow readers), 18 were recruited from the Salzburg longitudinal sample for the study of reading development (e.g., Ref. 32). These participants (exclusively males) repeatedly took part in studies of our group. Reading speed was assessed at all participations and the speed-deficit of the slow readers was confirmed by every assessment^32^. At the time of the current study, these participants were young adults (mean age: 17 *y*; see 31 for more details). The additional participants were adults who self-reported that they had been suffering from persistent reading difficulties since the beginning of their formal education and adult fluent reading controls (primarily university students; mean age: 24 *y*). We administered a paper-and-pencil reading speed test prior to the eye tracking study. The test required to silently read sentences and mark them as correct (e.g., “*A week has seven days*”) or incorrect (e.g., “*A weighing machine measures the height of a person”*). The incorrect statements were obvious violations of common knowledge and hence judging the correctness was easy (*M* < 1 incorrect marking in both groups). Thus, the measure (number of correctly marked sentences within 3 minutes) is an index of reading speed. The preliminary norms of the test are based on a sample of 309 University students. All slow readers exhibited a reading rate of less than percentile 16; all fluent readers had a reading rate greater than percentile 30.

The final selection of the participants relied on the reading speed of the sentences of the eye movement assessment. We converted the reading times of the sentences into a measure of words per minute (*wpm*). The criteria for inclusion in the group of the slow and the fast readers were reading rates of less than 180 and greater than 250 *wpm*, respectively. These criteria were fulfilled by all of the fast readers (*n* = 35) and by 17 (out of the 18) slow readers from the Salzburg longitudinal sample and by 15 (of 17) additional slow reading adults (final *n* = 32). The mean reading rate of the slow readers was less than half the rate of the fast readers with means of 138 *wpm* (*SD* = 32) and 303 *wpm* (*SD = 46)*, respectively. Impaired reading speed is the hallmark symptom of developmental dyslexia in regular orthographies (whereas reading accuracy and comprehension are preserved; Ref. 32, 33). Furthermore, the criterion that our slow readers suffered from persistent reading difficulties throughout their school careers is a diagnostic criterion for specific reading disorder (i.e., developmental dyslexia). However, we refer to our sample as slow readers, because some of them did not have a formal diagnosis of dyslexia. Moreover, in the recently revised diagnostic and statistical manual of mental disorders (DSM-5) developmental dyslexia is not a separate diagnosis anymore^34^. All participants had normal or corrected-to-normal vision. They gave an informed consent before inclusion in the study. The study was conducted in accordance with the national legislation for the protection of human volunteers in non-clinical research settings and the Declaration of Helsinki. The ethics committee of the University of Salzburg (“Ethikkommission der Universität Salzburg”) approved the experimental protocol of the study.

### Material

We presented the 144 sentences of the Potsdam Corpus^29^. Sentence length varied from 5 to 11 words (M = 7.9). The corpus comprises 1138 words (994 for analyses after omitting the sentence-initial word). Word predictability is expressed as the probability [*p*; range: 0–1] with which the participants of the norming sample correctly guessed the upcoming word on the basis of the preceding sentence context^29^. Word frequency are the *log*-transformed occurrences per million (range: 0.0–4.4) from the CELEX database^35^. Word length ranged from 2 to 20 letters (2 and 3 letter words were considered as single category as were words with more than 12 letters). Predictability was transformed into *logit*-units^29^. Word length was transformed into reciprocal values (1/number of letters). This made it possible to generate better fitting models (see below). Effects of word length and frequency are not reported in the current study (they were reported in Ref. 31).

### Procedure

Eye movements were recorded monocular from the participants' right eye with an *SR-Research* (Ontario, Canada) *EyeLink CL* eye tracker (sampling rate: 1 kHz). A 9-point calibration routine was administered before the presentation of 12 familiarization trials, repeated after familiarization and after a short break halfway through the experiment. The tracking error was kept below 0.5° of visual angle. Sentences were typed in mono-spaced, bold font (Courier New, 14 pt.) presented on a CRT monitor (1024 × 768 pixel resolution, 120 Hz refresh rate). The participant's head was stabilized by a chin and forehead rest. From the viewing distance of ~52 cm, a single character had a width of ~0.3°.

Prior to the presentation of a sentence, a fixation cross was presented near the middle of the left screen frame. After detecting a fixation (minimum duration: 100 ms) at the fixation cross, the sentences were presented in such a way that the participants centrally fixated the sentence-initial word. When the system did not detect a fixation at the fixation cross within 5 seconds the system was recalibrated. Participants read the sentence silently for comprehension. Looking at an “x” at the bottom right corner of the monitor terminated the trial and triggered the reappearance of the fixation cross. After 24 of the sentences the experimenter verbally presented a simple comprehension question.

### Data treatment and analyses

Saccades were identified with the *eyetrackR* package (version 0.16) in the R environment for statistical computing (version 3.1.1). Fixation durations were obtained with an in-house Perl script. We observed 70,635 and 33,192 fixations in the groups of the slow and fast readers, respectively. Fixations of less than 80 ms were discarded (<1% of the data). We analyzed the effect of predictability (of word *n* and *n + 1*) by linear mixed models (LMM) and generalized linear mixed models (GLMM) from the *lme4*-package (version 1.1–7; Ref. 36). LMM deal well with unbalanced designs (e.g., due to skippings) and estimate robust coefficients for correlated predictors. In the sentence corpus of the present study, word predictability was moderately correlated with word frequency (*r* = .30). Our dependent measures were skipping probability, single fixation probability, first fixation duration (FFD), single fixation duration (SFD) and gaze duration (GD). Skipping probability is the probability that a word did not receive a fixation during first-pass reading (i.e., during the first encounter of the word); skipped words may be fixated after they had been skipped initially (i.e., during second-pass reading). Single fixation probability and duration refers to instances in which a word received a sole fixation during first-pass reading (i.e., a fixation on a word was not considered a single fixation when the word had previously been skipped). FFD is the first fixation on a word during first-pass reading (regardless whether it was the single fixation on a word or the first of multiple fixations). GD is the sum of the duration of all fixations on a word during first-pass reading. FFD are sometimes considered as an “early” measure (because it reflects instantaneous effects), whereas GD is considered as a “late” measure (because it also reflects effects which occur later in visual word recognition; e.g., Ref. 37).

**Figure 1:**
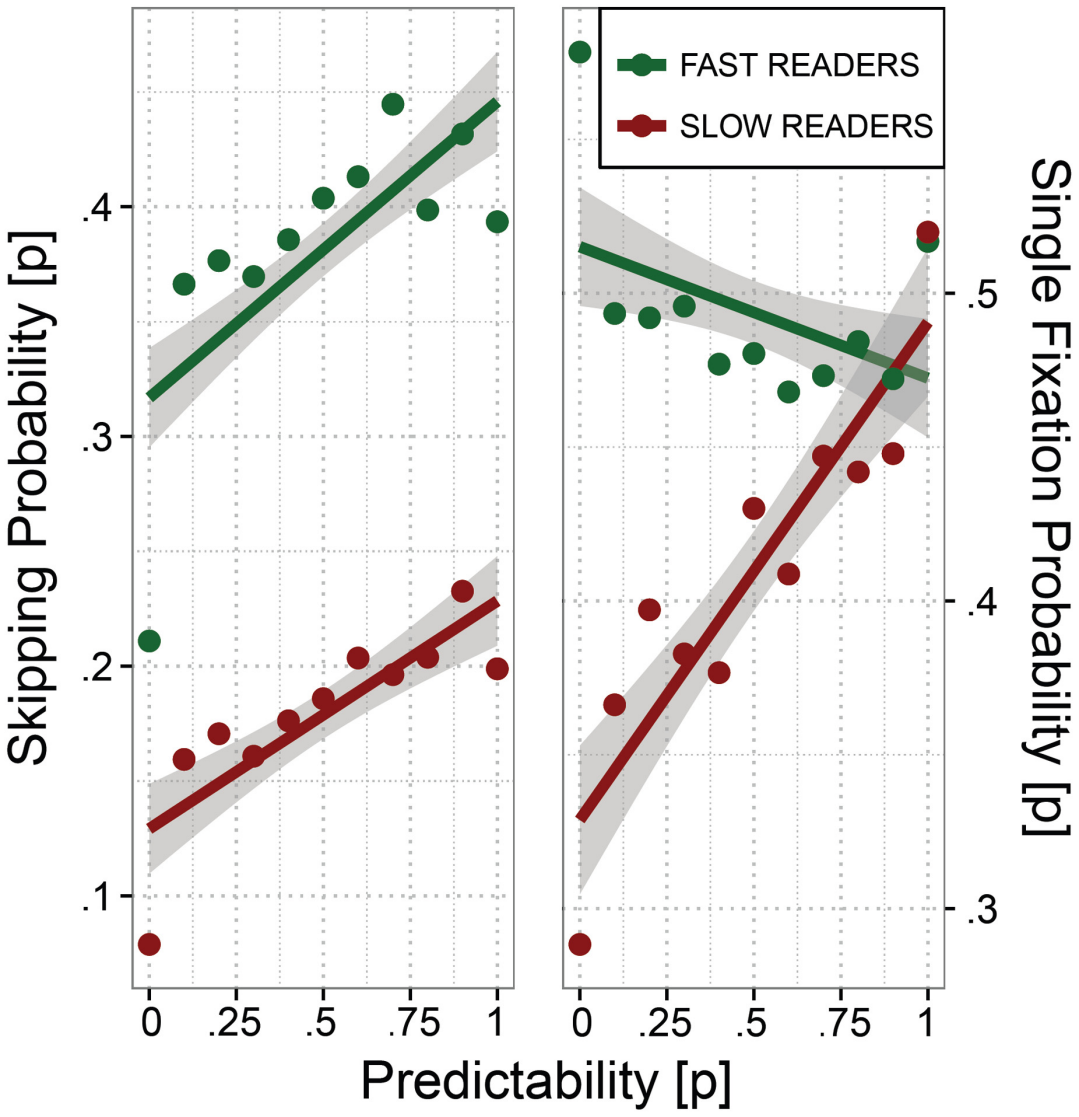
Skipping and single fixation probability in relation to word_n_ predictability for the fast and the slow readers. For the purpose of illustration, the predictability values [*p*] were summarized into 11 predictability categories (i.e. rounded to 1 decimal). The CI represents 1 SEM.

**Figure 2:**
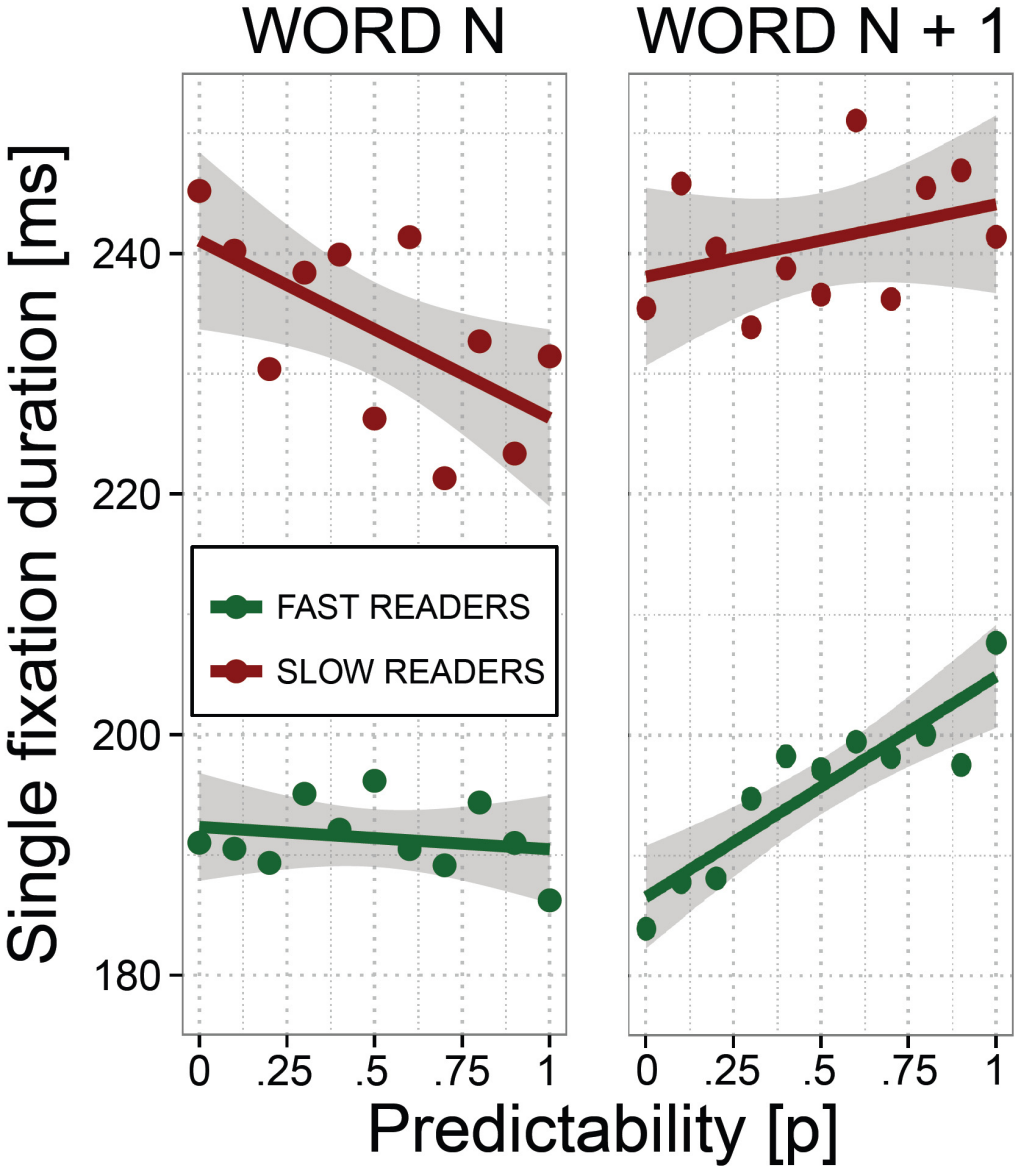
The effect of word predictability of word_n_ and word_n+1_ on single fixation duration of the fast and the slow readers. The CI represents 1 SEM.

Skipping probability and single fixation probability were analyzed with GLMM (i.e., logistic regression), because these probabilities are binary measures (the models' family was “binomial”; test statistic: Wald's *Z*). As predictors (i.e., fixed effects, *b*) we considered the group (fast versus slow readers) and the length, the predictability and the frequency of word_n_. We modeled the interactions between frequency X predictability and group X predictability. For the LMM analyses of first fixation duration (FFD), single fixation duration (SFD) and gaze duration (GD) we considered the group of readers (fast versus slow) and the length, the frequency and the predictability of word_n_ and the predictability of word_n+1_ as fixed effects. We modeled the interaction between group X predictability, group X frequency and predictability X frequency of word_n_ and group X word_n+1_ predictability. Note that differences between the fast and the slow readers in the word_n_ and word_n+1_ predictability effect will be reflected by interactions of predictability X group (with the fast readers as the reference group). The values of these interactions are coefficients (their sign indicating the direction of the group difference). Their significance is, similar to the other fixed effects, tested against zero (test statistic: *t*-value). SFD, FFD and GD were *log*-transformed (natural logarithm), because their distributions were considerably right skewed (Fig. 1 and Fig. 2, however, present our findings with untransformed data). Due to the log-transformation of the dependent measures (and due to modeling interactions of effects), the estimates of fixed effects are numerically very small. Thus, for an easier grasp of the fixed effects, we report them with a scientific notation (e.g. the value 0.00345 is reported as 3.45−3, i.e., the original value is obtained by 3.45*10^−3^). For the graphical depiction of the fixed effects we obtained 95% confidence intervals (CI) with the *confint*-function (of the *stats-*package; version 2.15.3). As random effects on the models' intercept we considered participants, the sentences and the individual words.

## Results

### Comprehension and descriptive measures

The means of correct answers to the 24 comprehension questions was greater than 23 in both the slow and the fast readers (*M* = 23.16 and 23.57, respectively; *min* = 21 and 20). Despite the close-to-ceiling performance in both groups, a Wilcoxon rank sum test revealed a significant group difference (*W* = 419). This significant difference was due to the fact that 25 (~71%) of the fast readers, but only 16 (i.e., 50%) of the slow readers answered all 24 comprehension questions correctly. An inspection of the erroneous answers of the slow readers revealed that the majority committed a specific error for one particular sentence (see Discussion). Table 1 provides descriptive measures of fixation probabilities and measures of processing time of the 994 words of the sentence corpus. As evident from the Table, the slow readers exhibited much smaller probabilities than the fast readers for word skipping and single fixations. Furthermore, all measures of processing times were longer in the slow than in the fast readers; the group difference was particularly pronounced for GD (*M*_diff_ > 160 ms).

### Costs of skipping and the effect of predictability on skipping rate and single fixation probability

We compared the GD prior to word skips with the GD when the upcoming word was fixated. The means of the fast readers were 226 and 216 ms, respectively; the means the slow readers were 413 and 379 ms. Thus, we found a small “cost” of skipping in the fast readers (*M*_diff_ = 10 ms; *t*_34_ = 3.8); and a larger “cost” in the slow readers (33 ms; *t*_31_ = 3.7). The group difference in the size of the effect was significant (*t*_65_ = 2.6). Next we assessed the effect of predictability on the skipping rate of the fast and the slow readers; presented in the left panel of Figure 1. Increasing word predictability led to an increase in skipping probability (*b* = 1.75−1, *SE* = 1.90−2, *Z* = 9.2) similarly in both groups (group by predictability: *b* = 1.68−2, *SE* = 1.05−2, *Z* = 1.6). The right panel of the Figure shows the single fixation (SF) probability in relation to predictability. In the group of the fast readers, SF probability decreased with increasing predictability of the words (because they frequently skipped predictable words; *b* = 3.36−2, *SE* = 1.77−2, *Z* = 1.9). For the slow readers, the SF probability steeply increased with predictability resulting in a significant interaction of group by predictability (*b* = 2.65−2, *SE* = 8.39−3, *Z* = 32).

### The effects of predictability on fixation and gaze duration and its relation to reading rate

**Figure 3:**
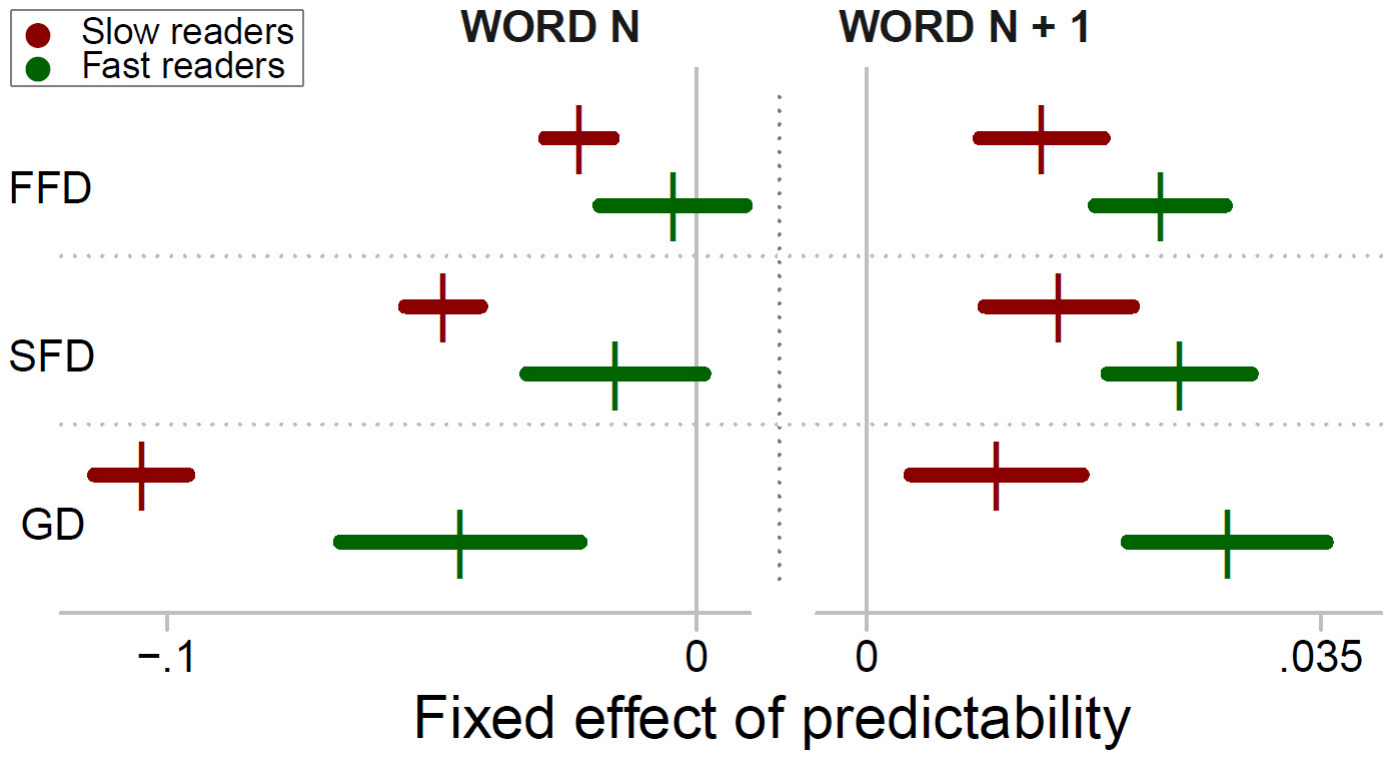
This coefficient plot shows the fixed effect (vertical bars) of *logit*-predictability on the *log*-transformed first fixation duration (FFD), single fixation duration (SFD) and gaze duration (GD) for word_n_ and word_n+1_ of the slow and the fast readers. Fixed effects left of the vertical midline (i.e., with a negative sign) indicate facilitation by predictability. Fixed effects to the right of the midline indicate increasing duration of the measures with increasing predictability. The horizontal bars represent the 95% CI. If the CI includes 0 (i.e., if it crosses the midline), then the effect is non-significant.

Figure 2 depicts the effect of predictability of word_n_ and word_n+1_ on SFD. As evident from the left panel of the Figure, the fast readers exhibited little effect of word_n_ predictability, whereas the slow readers exhibited increasingly shorter SFD with increasing predictability. The right panel of Figure 2 shows that the fast readers exhibited increasingly longer SFD with increasing predictability of word_n+1_. This effect was less pronounced in the slow readers. The fixed effects of word_n_ and word_n+1_ predictability on FFD, SFD and GD are shown by a coefficient plot in Figure 3. As evident from the Figure, the slow readers exhibited reliable facilitatory effects of word_n_ predictability for every measure, but the effect was particularly pronounced for GD. For the fast readers, word_n_ predictability exerted a reliable facilitatory effect only for GD. Their effects of word_n+1_ predictability, in contrast, were reliable for each measure: Their FFD, SFD and GD on word_n_ were increasingly longer with increasing word_n+1_ predictability. Within the group of slow readers, this effect was markedly less pronounced. The group differences of the effect of word_n_ and word_n+1_ predictability were all significant (group by word_n_ and group by word_n+1_ predictability: all *t*s > 3.2). Put differently, we found significantly higher facilitation of word_n_ predictability, but significantly lower effects of word_n+1_ predictability in the group of slow readers compared to the fast readers.

**Figure 4:**
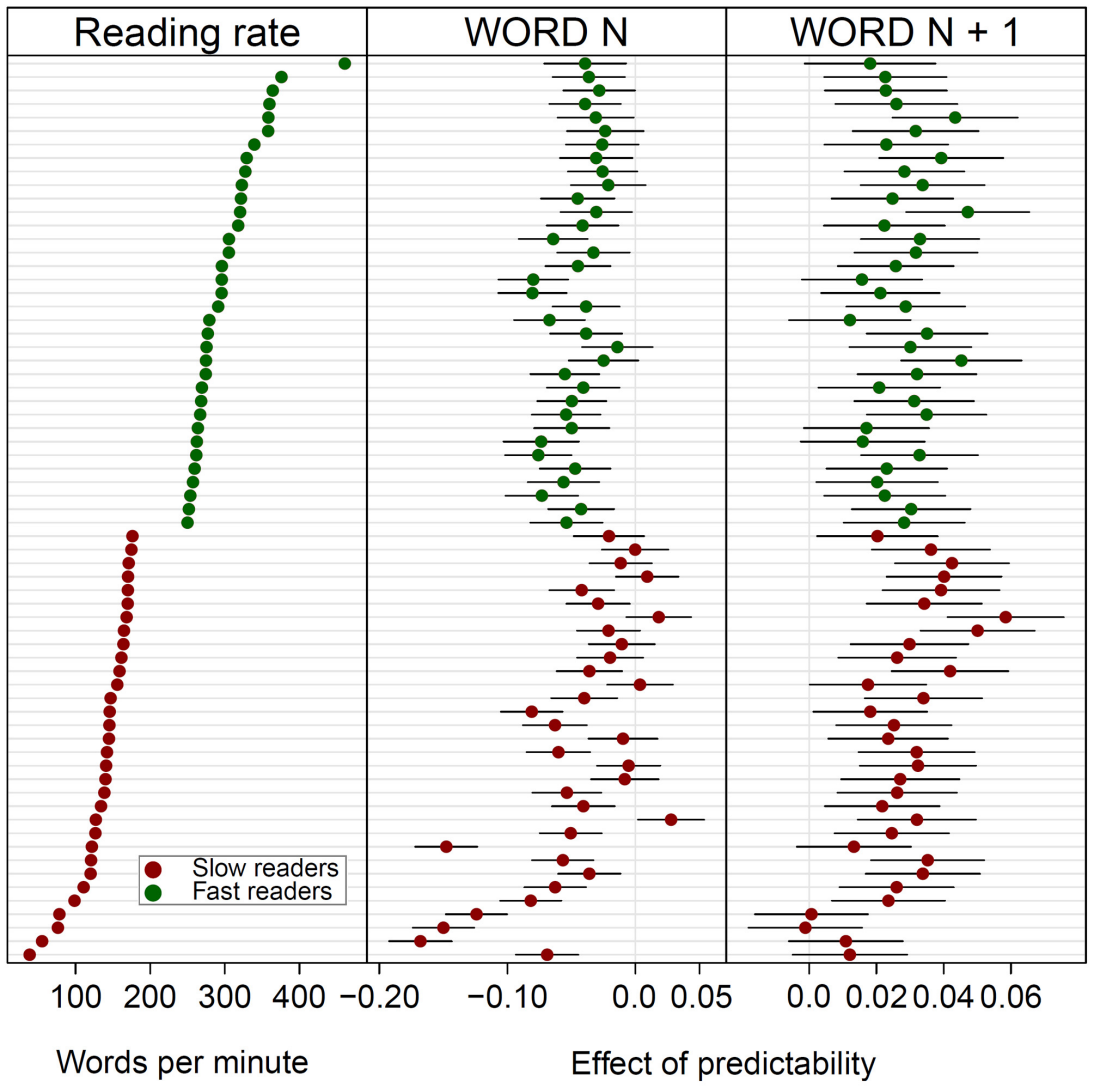
“Caterpillar plots” of the reading rate and the effects of word_n_ and word_n+1_ predictability on gaze duration (GD) of the slow and the fast readers. The vertical bars represent the 95% prediction intervals. Fixed effects with a negative sign (i.e., left of the vertical mid-line) indicates decreasing GD with increasing predictability; fixed effects with a positive sign indicate increasingly longer gaze duration with increasing predictability. Participants are ordered by reading rate.

Next we looked at the association of the effects of predictability with the reading rate of the participants. To this end, we estimated the individual effect of predictability (on GD) by inserting a random slope for the fixed effect of predictability in the LMM. The “random” slope expresses the magnitude with which the slope of the effect of an individual participant deviates from the mean slope of the whole group. The findings are depicted by “caterpillar plots” in Figure 4. The left panel shows the individual reading rate (in words per minute; *wpm*) of the fast and the slow readers (sorted – top-to-bottom – in descending order from the fastest to the slowest reader). The middle and the right panels show the individual effects of the predictability of word_n_ and word_n+1_, respectively. It is evident from the Figure that, in the group of the fast readers, increasing predictability of word_n_ led to shorter GD. The fastest readers, however, exhibited the least facilitation (i.e., the smallest fixed effects). The correlation of reading rate with the fixed effect of word_n_ predictability was, among the fast readers, moderate (Pearson's *r* = .44). In the group of the slow readers this correlation was much higher (*r* = .72): The slowest readers exhibited the most pronounced facilitation by word_n_ predictability. The left panel of Figure 4 reveals that not a single one of the fast reader exhibited a negative slope of word_n+1_ predictability. Put differently, for each of the fast readers the LMM estimated increasing GD with increasing predictability of the upcoming word. Furthermore, Figure 4 indicates that there is no notable correlation between word_n+1_ predictability and the individual reading rate of the fast readers (*r* = .01). Within the group of the slow readers, in contrast, we observed a strong correlation of reading rate with word_n+1_ predictability (*r* = .69) in the direction that the readers with the most aggravated speed deficit exhibited the smallest effects of word_n+1_ predictability.

### The time course of the predictability effect

**Figure 5:**
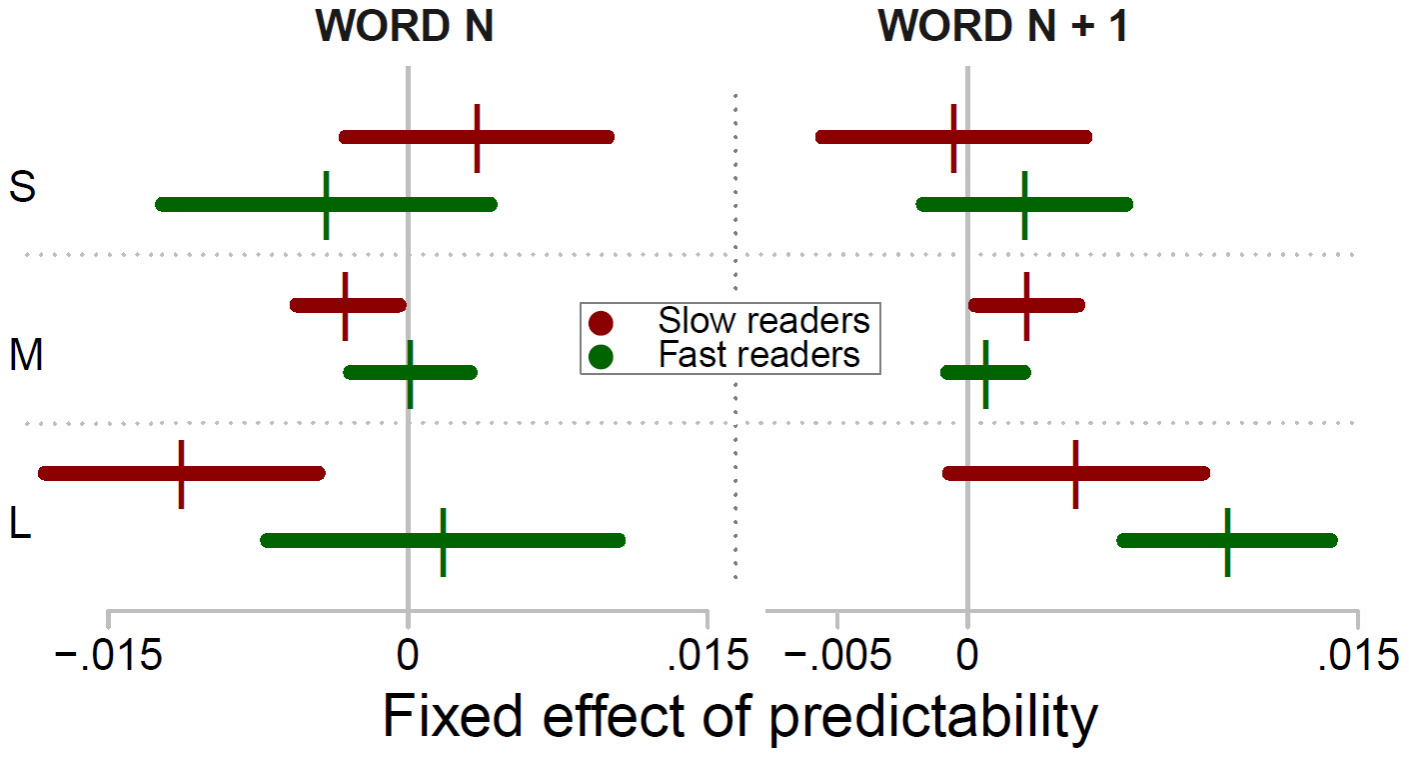
This coefficient plot shows the fixed effects (vertical bars) of predictability of word_n_ and word_n+1_ for short, medium and long first fixation duration (S, M and L, respectively) of the fast and the slow readers. The horizontal bars show the 95% CI of the effects. Fixed effects to the right of the vertical midline indicate increasing fixation duration with increasing predictability.

We assessed when predictability exerts its effect. To this end, we distinguished – individually for each participant – between the 33% shortest FFD (i.e., percentile < 33), FFD between the percentiles 33 and 66 and the 33% longest FFD (percentile > 66). We refer to the categories as short, medium and long FFD (S, M and L in Fig. 5). The group specific means of the categories were 138, 182 and 251 ms for the fast readers, and 151, 216 and 318 ms for the slow readers, respectively. Separately for each of the categories, we repeated the analyses of the predictability effect. The rationale of the analysis is that an early effect of predictability would be more evident in short than in long FFD. Conversely, a late effect of predictability would be more pronounced in long FFD^38^,^39^. The fixed effects of the predictability of word_n_ and word_n+1_ are displayed by the coefficient plot in Figure 5. As evident from the left panel of Figure 5, the fast readers exhibited no reliable effects of word_n_ predictability. The direction of the effect was from a negative slope (i.e., indicating facilitation, if it were significant) for short FFD towards a positive slope for long FFD. The important finding here is that the slow readers exhibited a time course of word_n_ predictability in the opposite direction: They exhibited increasingly stronger effects towards facilitation from short to long FFD. Accordingly, a LMM with type of FFD (i.e., short to long) as additional fixed effect revealed a significant three-way interaction between word_n_ predictability, type of fixation and group (*b* = −8.79−3, *SE* = 1.83−3, *t* = 4.8). The right panel of Figure 5 depicts the effect of the predictability of word_n+1_. It is evident from the Figure that word_n+1_ predictability wielded its influence on long FFD. Moreover, the effect was stronger in the fast readers (*b* = 9.98−3, *SE* = 1.57−3, *t* = 4.9) than in the slow readers (group by word_n+1_ predictability: *b* = −5.81−3, *SE* = 2.49−3, *t* = 2.3).

## Discussion

The main objective of the present eye movement study was to assess to what extent fast and slow reader exhibit evidence for generating *forward inferences* during reading. The generation of forward inferences, a term adopted from the predictive coding framework^10^,^11^, refers to predicting upcoming words. Accordingly, we assessed the relation between various eye movement measures with word predictability. The measures were the probability with which words are skipped or processed with a single fixation and the duration of first fixations, single fixations and gazes (FFD, SFD and GD). We examined effects of the predictability of the current (word_n_) and of the upcoming word (word_n+1_). Moreover, we assessed the relation of the individual effects of word predictability with the reading rate of our participants and examined the time-course of the predictability effect.

In brief, our findings indicate that readers generate forward inferences about the probable identity of upcoming words, but slow readers do so to a smaller extent than fast readers. In the latter group, the individual effects of word predictability were strongly associated with reading rate: The most speed-impaired readers exhibited the strongest facilitation by the predictability of the currently fixated word, whereas they exhibited the least effect of the predictability of the upcoming word. Moreover, the effect of predictability emerged late in the slow readers. They exhibited the strongest effects of word_n _predictability for the measure of GD (a measure which captures late effects; Ref. 37) and the effect was more expressed in long compared to short FFD. In the group of the fast readers, we found that a word's predictability has a reliable effect before the word is encountered (i.e., the effect of word_n+1_ predictability). This finding shows that, in fast readers, predictability exerts an early influence during silent reading. However, the influence of the predictability of word_n+1_ was only evident in long FFD (on word_n_). This finding indicates that generating forward inferences about the identity of upcoming words occurs when the processing of the currently fixated word is well-advanced. Now we proceed to a detailed discussion of these findings and their implications.

The first indication that an upcoming word can influence processing times before it is encountered was provided by the effects of predictability on measures of fixation probability. In line with a wealth of previous research (reviewed, e.g., in Ref. 26), predictable words were more often skipped than unpredictable words. The influence of predictability on skipping probability was of similar magnitude in our fast and slow readers. However, the skipping rate of the slow readers was, in general, extremely low (13%; fast readers: 30%). Even for the most predictable words, the slow readers exhibited a skipping probability of only about 20% (fast readers: ~40%). We consider this as a first indication that predicting upcoming words is impaired in slow readers. With regard to processing times prior to skips, both groups exhibited prolonged GD (i.e., while their eyes were on word_n−1_). Put differently, we observed costs of skipping (a controversial finding in the literature of eye movement control during reading, e.g., Ref. 27, 40). This cost was substantially higher in the slow (~30 ms) than in the fast readers (~10 ms). In the light that the average GD of our slow readers were substantially longer than those of the fast readers (~160 ms), we consider this as a first indication that the time course of generating forward inferences differs between readers: It is considerably delayed in slow readers.

A further indication that predictability had a strong, but comparatively late effect in the slow readers is that their probability of recognizing a word with a single fixation steeply increased with increasing predictability of word_n_. For the most predictable words (which were very often skipped by the fast readers), the slow readers exhibited a single fixation probability which was similar to the average single fixation probability of the fast readers (i.e., for all words regardless of predictability). In a nutshell, the slow readers processed those words with a single fixation which the fast readers skipped. This finding indicates that, in the slow readers, predictability exerted its effect often too late to warrant word skipping, but facilitated word recognition when the word was fixated. Accordingly, we observed reliable facilitatory effects of word_n_ predictability on the FFD, SFD and GD of the slow readers. The effect was least pronounced for FFD and most pronounced for GD. This pattern also supports the notion that the predictability effect emerges late in the slow readers, because FFD is a sensitive measure for early effects, whereas GD also captures late effects^37^.

The notion of a previous study^30^ that the difference between highly proficient and less proficient readers is merely “one of degree” does not concur with the substantial group differences we found concerning the effects of predictability as well as the strong association of the predictability effect with the reading rate of our slow readers. A possible reason that the previous study found comparatively little differences with regard to word predictability is that the study compared high-proficiency readers with average-proficiency readers. We compared fast readers and readers with serious speed impairment. Indeed, it were the readers with the most severe reading speed impairment which exhibited the most pronounced (facilitatory) effect of word_n _predictability - accompanied by a comparative absence of an effect of word_n+1 _predictability. The profound group difference in the capability (or the automaticity) of generating forward inferences during reading between slow and fast readers, would explain a seemingly paradox finding of the protagonists of the *lexical quality hypothesis*^41^. In one of their experiments short stories were presented. The presentations were unpredictably halted and the participants (children differing in reading skill) had to guess the continuation word of the last and uncompleted sentence. The authors summarized the findings as follows (p. 281):

*“The paradox is that less skilled readers *[...] *are not as good as skilled readers at producing contextually constrained words. *[...] *skilled readers predicted word targets significantly more accurately than less skilled readers. This accuracy advantage included exact target prediction, not just contextually appropriate nontargets.”*

However, the interpretation that predictive coding (i.e., the generation of forward inferences) plays a significant role in reading and that slow readers are less capable to predict upcoming words does *not* imply that *guessing* upcoming words would a be valuable reading strategy (see below). The view of reading as a “psycholinguistic guessing game” (e.g., Ref. 3) has been refuted (and rightly so; Ref. 42). A plausible account of how forward inferences wield influence in reading is as follows. As mentioned in the Introduction, predictive coding relies on two different mechanisms. First, predictions can be based on prior experience (e.g., Ref. 13). Second, predictions can arise from the extremely rapid processing of the low spatial frequencies (LSF) of a visual percept. This partial information is rapidly transmitted to the orbitofrontal cortex where predictions are generated. These predictions are transferred – top-down – to brain regions in the inferior temporal cortex where they are integrated with the slower arriving, but more detailed bottom-up information^14^. In reading both these mechanisms probably interact^43^. On the one hand, the sentential context (i.e., the prior experience) makes – in some instances – predictions possible. Parafoveal preprocessing, on the other hand, provides coarse visual information about the upcoming word. The more constraining the preceding sentence context is, the more visual information about a parafoveal word is utilized for narrowing down the set of potential continuation words^43^. Notably, such an interaction of predictability and parafoveal preprocessing is suggested by the SWIFT model^40^, a computational model of eye movements control during reading. The model's engine for visual word recognition does not (always) require that a word is fully processed – an assumption which is particularly relevant for word skipping. A recent study indeed showed that the processing of words, which are skipped during first pass reading, is more shallow than the processing of fixated words (Ref. 44; but see also Ref. 27).

Supposedly, generating a prediction of upcoming words and align it with the parafoveal visual percept of the word must occur timely well synchronized to prevent (an overabundance of) prediction errors which would hinder reading more than it would accelerate the process. We suppose that in slow readers the orchestration of the two processes is “out of tune” due to a mismatch in the timing of bottom-up and top-down processing. An illustrative demonstration of this assumption is provided by the erroneous answer which many of the slow readers (12/31, i.e., >30%) gave to one of the comprehension questions. The question was “*What should be read?*”. The sentence was “*Lies mir bitte die **Angaben** vor*” [approx. translation: *Please read me the specifications*]. The erroneous answer always was “*die **Aufgaben***” [*the instructions*] which would be a plausible continuation of the preceding sentence context. Note that the presented word and the erroneous response are visually similar. However, they start with a different phoneme. The initial phoneme of the one word is/a/(as in ***a**rt*); the other word starts with the diphtongue /aʊ/ (as in *h**ow***). Thus, they slow readers, who committed the error, generated an incorrect prediction; bottom-up processing was too slow to correct the error. To become aware of the false prediction, bottom-up processing would only have required accessing the phonology of the very beginning of the word. (That phonological codes are activated early – even during silent reading – has been shown for unimpaired readers; e.g., Ref. 45). Thus, the error is telling with regard to the slow reader's timely ill-aligned orchestration of bottom-up and top-down processing. Noteworthy, speed-impaired access to phonology is considered as a potential core deficit of disordered reading (i.e., developmental dyslexia; Ref. 33, 46).

We can only speculate on the cause-and-effect relationship between the, compared to the fast readers, limited generation of *forward inferences* during reading and the speed-impaired bottom-up processing in slow readers. An interpretation in terms of causality would require an experimental study with beginning readers. However, we confirmed a core assumption of the lexical quality hypothesis, that is, that fast readers efficiently process even the most unpredictable words (i.e., mostly by a single fixation). To illustrate, the fast readers singly fixated approx. 60% of the words which had a predictability of *p* = 0. The slow readers, to the contrary, recognized less than 30% of these words with a single fixation. Thus, in fast readers word recognition is evidently effortless even when the processing of a word must proceed bottom-up. For the slow readers, to the contrary, this process is more effortful and much more time consuming. Thus, it is plausible that the difficulties with processing the currently fixated word prevent preprocessing the upcoming word. This interpretation coincides with the *foveal load* hypothesis^47^ and the notion of a *dynamic perceptual span*^48^. In brief, the perceptual span is the effective field of vision from which information is extracted during a fixation^49^. The span is *dynamic* which means that its size is adjusted depending on the difficulty of the currently fixated word (i.e., the *foveal load*; Ref. 47). The perceptual span of slow readers may be, on average, smaller than that of a fast reader due to their frequent difficulties with visual word recognition (i.e., they need to devote more of their attentional resources to the foveal words). A small span would prevent obtaining (coarse) visual information from the upcoming word which would support the generation of forward inferences by interacting with context-based predictions^50^. It has been shown that beginning readers have a smaller perceptual span than skilled (adult) readers^51^,^52^ and there is recent evidence that, during reading, dyslexic readers^53^ and less experienced readers^54^ obtain less parafoveal information than unimpaired, experienced readers.

Another explanation for the comparative lack of forward inferences in slow readers could be that they suffer from a visual impairment or from deficient visual attention. The studies on predictive coding in object recognition revealed that the coarse visual information (i.e., the LSF) of the perceived object is transmitted rapidly to the orbitofrontal cortex by magnocells (M-cells; nerve cells with thickly myelinated axons with a high transmission speed). It was hypothesized that a reading disorder is caused by a specific impairment of this cell type which is also crucially involved in visual attention. The theory is known as the *magnocellular theory of dyslexia*^55^. A deficit of the M-cells of the visual system in slow readers may compromise the transmission of LSF information to the orbitofrontal cortex. Supporting evidence for this notion was provided recently^56^. The study showed that Chinese dyslexic children performed inferior to normal reading children in a Chinese character recognition task. This was especially the case in a condition in which the visual image of the characters was spatially filtered so that processing required the LSF sensitivity of the M-cells. However, we note that the magnocellular theory of dyslexia and visual-attentional deficits in dyslexic readers are discussed controversially (M-cell theory: e.g., Ref. 57; attention: e.g., Ref. 58).

Finally, a reconciliation of the lexical quality hypothesis (which supposes little influence of context-based predictions in reading) with the predictive coding framework could be as follows. As postulated by the lexical quality hypothesis, a tight coupling of the semantics of a word, its orthography and its phonology is the bedrock of fast and efficient reading. It is entirely conceivable that generating a prediction of upcoming words and finding it confirmed by the visual input plays a crucial role for establishing such tight connections in the triangle of meaning, phonology and orthography which characterizes *high-quality* lexical representations (6). From this perspective, generating predictions about probably upcoming words during reading acquisition could be a causal factor in forming high-quality representations. A recent intervention study provided evidence for this suggestion. The study reported that (pre-)activating the semantics of to-be-learned words helped beginning readers with the acquisition of stable associations between the phonological and the orthographic properties of words^59^. Whether the comparative lack of generating forward inferences in slow compared to fast readers is a symptom of a reading disorder or whether it plays a more direct and causal role in accounting for the speed impairment of slow readers is subject to future studies. As a concluding remark, we believe that investigating reading from the perspective of predictive coding promises to shed new light on this intriguing feat which seems so effortless for most of us, but which is a matter of continuous struggle for many.

## Author Contributions

S.H. wrote the manuscript. S.H. and S.S. analyzed the data. S.S. prepared the figures. B.G. performed the experiment. F.H. made important theoretical contributions. All authors reviewed the manuscript.

## Figures and Tables

**Table 1:** Means and standard errors of eye movement characteristics of the fast and the slow readers and the corresponding *t*-values of the group comparisons

	Fast readers		Slow readers		*t*-value^a^
	*M*	*SE*	*M*	*SE*	
*Fixation probabilities[p]*					
Skipping	.30	.013	.13	.011	10
Single fixations	.53	.012	.34	.018	9.0
Multiple fixations	.17	.009	.53	.023	15
*Durations [ms]*					
First fixation	190	4.2	228	4.4	7.5
Single fixation	191	2.9	238	4.8	8.6
Gaze	222	3.4	386	22.9	7.4

*Note*. ^a^*df* = 65.
